# Presentation of systemic lupus erythematosus (SLE) in emergency department: a case report

**DOI:** 10.1186/1756-0500-6-181

**Published:** 2013-05-05

**Authors:** Natália Fernandes, Guilherme Gomes, Carlos Capela

**Affiliations:** 1 Department of Internal Medicine, Hospital of Braga, Braga, Portugal; 2 Life and Health Sciences Research Institute, School of Health Sciences, University of Minho, Braga, Portugal; 3 Autoimmune Diseases - Department of Internal Medicine, Hospital of Braga, Braga, Portugal

## Abstract

**Background:**

Abrupt and life-threatening presentations in connective tissue diseases (CTD) are rarely reported. Their early recognition and specific management could change course disease. SLE is a multisystem inflammatory disease that is often difficult to diagnose in the emergency department (ED).

**Case presentation:**

A 26-year-old woman presented to the ED with a 48 hour history of progressive dispnea, generalized edema and left lower chest pain with non-productive cough. On examination, patient was feeling very ill, afebrile, tachycardic, tachypneic and a peripheral oxygen saturation of 94% on 40% supplemented oxygen with raised jugular venous pressure was noted. Intermittently, she presented an obtunded state of consciousness. A large pericardial, pleural and abdominal effusion was confirmed and a broad differential diagnosis was made. The patient had a 6 months history of inflammatory polyarthralgias involving initially interphalangeal joints, evolving, sometime later, the knees and elbows bilaterally and she was started glucocorticoids. 12 days before admission, she had had symptoms of a urethritis episode. In the context of an immunosupressed patient, with initial focal urologic complains, evidence of multiorgan dysfunction and a picture resembling a distributive shock, dictated a low threshold for sepsis.

**Conclusions:**

Separating an acute episode of SLE from sepsis, on emergency grounds, can even be the most challenging decision. In the ED, acute life-threatening and multisystemic conditions should arise the suspicion of autoimmune diseases, particularly SLE.

## Background

Abrupt and life-threatening presentations in connective tissue diseases (CTD) are rarely reported. Their early recognition and specific management could change course disease. SLE is a multisystem inflammatory disease that is often difficult to diagnose in the emergency department [1]. To the emergencist, it`s important to consider SLE as possibility, when faced with a patient with symptoms and/or signs suggesting a multisystem disorder. Emergent complications of SLE are managed in the usual manner the most described being: strokes, acute myocardial infarctions, hemoptysis, respiratory distress and pulmonary emboli [2-4]. Other complications, such as pericardial tamponade [1], pulmonary hemorrhage, renal failure, or cerebritis should be managed with appropriate subspecialist consultation.

## Case presentation

**Figure 1:**
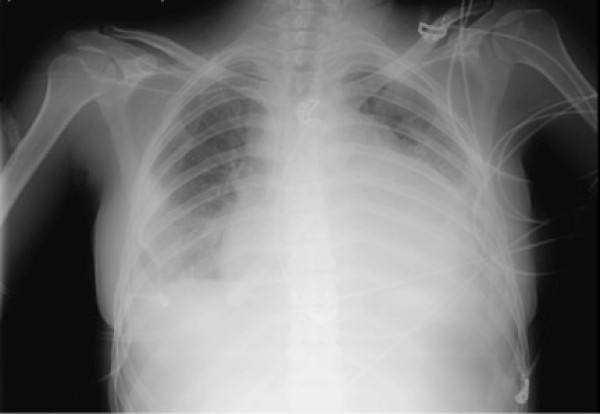
Chest roentgenogram showing cardiomegaly and right pleural effusion.

**Figure 2:**
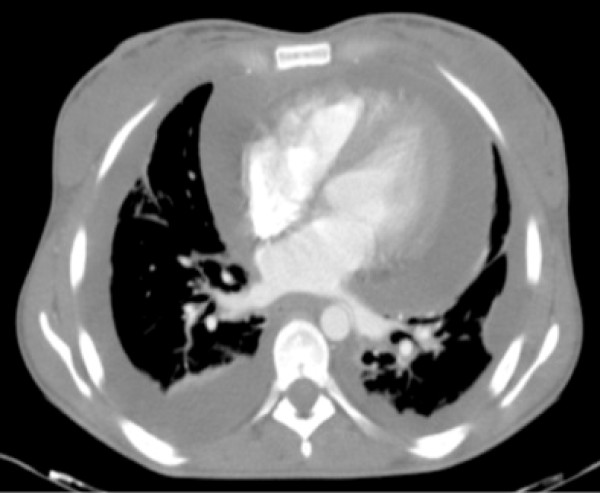
CT scan showing pleura-pericardial effusion.

**Figure 3:**
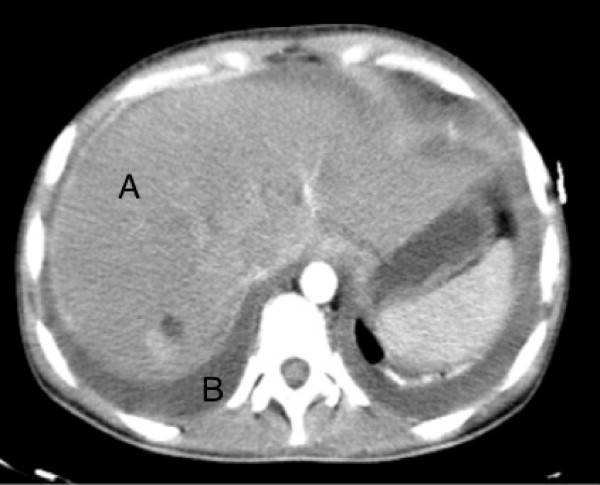
**An enlarged liver and ascites.** CT scan showing an enlarged and homogeneous liver (**A**) and ascites (**B**).

A 26-year-old Portuguese woman presented to the emergence department with a 48 hour history of progressive dispnea, generalized edema and left lower chest pain with non-productive cough. The patient had a 6 months history of inflammatory polyarthralgias involving initially interphalangeal joints, evolving, at some time later, the knees and elbows bilaterally. After ongoing rheumatologist evaluation, she started prednisolone 10 mg qd and hydroxychloroquine 400 mg qd, while waiting laboratory results. 12 days before admission, she had had symptoms of a urethritis episode, associated with unexplained anorexia and asthenia, which led her to suspend the therapeutic, even against medical advice. On examination, patient was feeling very ill, afebrile, with tachycardia (144 bpm), tachypnea (34/min), blood pressure 143/82 mmHg, peripheral oxygen saturation of 94% on 40% oxygen and raised jugular venous pressure. Intermittently she presented an obtunded state of consciousness. Aside a diffuse moderate edema and a livedoid skin discoloration, an erythematous rash over the cheeks and nasal bridge was noted. On auscultation heart sounds were found to be diminished and diffuse coarse crackles were noted on both lungs, with depressed vocal transmission in right basal thorax. Abdominal examination revealed moderate hepatomegaly and ascites. The electrocardiogram showed atrial fibrillation and low voltage QRS complexes. On chest roentgenogram (Figure 1) cardiomegaly and right pleural effusion were evident. Initial arterial blood gas showed metabolic acidosis (pH-7.134, PCO2-18 mm Hg, PO2-85 mm Hg, and HCO3-6 mEq/L). Intravenous fluid replacement was started, blood and urine were sent for culture and treatment was started with empiric antibiotics (ceftriaxone 2 g iv id plus ciprofloxacin 400 mg iv bid) and methylprednisolone 1 g iv bolus for 3 days. Patient was admitted to a high-dependency unit (HDU) for further evaluation, monitoring and support. An ultrasound of the heart showed a large circumferential pericardial effusion without signs of tamponade, which to the consulted assistant cardiologist merit only monitoring. Complementary CT scan (Figures 2 and 3) excluded pulmonary embolism or other thrombotic events, revealed an enlarged and homogeneous liver and confirmed pleuropericardial effusion and ascites. The laboratory survey revealed a white blood cell count (WBC) of 5.300 cells/mm3, with 70% polymorphonucleated cells (PMNs), a platelet count of 417.000 per mm3 and hemoglobin 7,8 g/dL. Her INR was 2.4. An extended metabolic and liver panel demonstrated: sodium 131 mmol/L, BUN 38.4 mg/dL, creatinine 1.4 mg/dL, total bilirubin 0.62 mg/dL, AST 401 U/L, ALT 85 U/L, and alkaline phosphatase 128 U/L. Ammonia was 38 μmol/L. C reactive protein 160 mg/l and ESR 87 mm/h. Urine evaluation demonstrates > 50 leucocytes and erythrocytes per high-power field, associated with proteinuria > 75 mg/dL Meanwhile, an immunologic panel, done 1 month earlier, was sent to us, showing: polyclonal hypergammaglobulinemia; positive ANA (1:160), anti-dsDNA antibodies (75 UI/mL) with positivity to anti SSA, SSB and Histone. Pleural fluid analysis showed an exsudate with no bacteria on a Gram stain. By the 2nd HDU day patient was feeling much better with remarkable edema regression. Blood gas normalized. The microbiologic results of the blood, pleural fluid and urine cultures became negative and antibiotherapy was suspended. Immunological panel revealed frank hypocomplementemia (C3 <1 UI; C4 – 9 UI; CH50 <10 UI); ANA (1:320), anti-dsDNA antibodies (175 UI/mL) with persisting positivity to anti SSA, SSB and Histone, as well as hypergammaglobulinemia and a direct positive Coombs test. A 24 hour urine collection demonstrates 2,1 g proteins. The patient was discharge by the 10th hospital day, on a therapeutic regimen of 60 mg/day of prednisolone plus hydroxychloroquine 400 mg/day, after an arrangement with the nephrologic clinic to eventually proceed to a renal biopsy.

## Conclusions

In this patient, SLE diagnostic criteria were accomplished when malar rash, polyserositis and positive immunology were summed to the previous described anamnestic features. All clinical and laboratory parameters pointed to a very active clinical process. As so, a true flare or a first full presentation of SLE were both tentative hypothesis, considering the short duration of the disease since she first call for medical attention and voluntary suspension of therapy. Such an explosive clinical presentation, in the context of an immunosupressed patient, with initial focal urologic complains, evidence of multiorgan dysfunction and a picture resembling a distributive shock, dictated a low threshold for sepsis and the decision to treat with antimicrobials until infection was surely excluded. This option was supported by generalized knowledge that, in sepsis, the prognosis varies with the elapsed time and the precocity and aggressivity of the initial measures. Separating an acute episode of SLE from sepsis, on emergency grounds, can even be the most challenging decision. We have also seen such a dramatic clinical picture in young people, particularly women, with catastrophic antiphospholypid syndrome, which led us to consider it in this patient. In retrospect, the full immunossupression with glucocorticoids aborted the potential negative consequences of the active SLE, and allowed a conservative approach to a potential lifethreatning consequence of a large pericardial effusion [5,6]. It remain unanswered if it was a real flare or the full expression of a progressing lupus, and perhaps it would turn an academic question in face of pressing clinical situations like this one, where treating with a structured reasoning, considering acute presentations of connective tissue disease and its complications will be more important and rewarding.

In the emergency department, acute life-threatening and multisystemic conditions should arise the suspicion of autoimmune diseases, particularly SLE. Circulating immune complexes and autoantibodies with multiples specificities, diffusely deposit and attack a multitude of organic targets, activating complement and mounting a full inflammatory response, provide a substract for the proteiform clinical e laboratorial expression of this disease. Usually, albeit impressive and frightening, the different manifestations can be successfully managed by adequate, early and full immunossupression [7].

## Consent

Written informed consent was obtained from the patient for publication of this case report and any accompanying images. A copy of the written consent is available for review by the Editor-in-Chief of this journal.

## Competing interests

The authors declare that they have no competing interests.

## Authors’ contribution

NF and CC treated the patient and wrote the case report; GG and CC supervised the writing and made some major changes in manuscript after reviewing the first versions. All authors read and approved the final manuscript.
